# Healthcare assessment tools—a valid source of data for pre-hospital care? Usability for ensuring medical care in the event of a disaster

**DOI:** 10.3389/fpubh.2024.1409371

**Published:** 2025-02-05

**Authors:** Robert Konrad, Denise Schuster, HJ Heppner

**Affiliations:** ^1^Institute of Rescue, Emergency and Disaster Management (IREM), Technical University of Applied Sciences Würzburg-Schweinfurt (THWS), Nuremberg, Germany; ^2^Department of Internal Medicine I, Faculty of Health, University of Witten/Herdecke, Witten, Germany; ^3^Chair of Internal Medicines (Geriatrics), Friedrich-Alexander University Erlangen-Nuremberg, Nuremberg, Germany

**Keywords:** care assessment tools, operational planning, resource allocation, vulnerable groups, health data

## Abstract

**Introduction:**

Providing care for vulnerable population groups in the event of a disaster or evacuation is in the interests of those affected, of local authorities, health insurance companies, other insurance companies and the responsible authorities and organizations with security tasks (BOS). Evacuation and supply planning is currently mostly carried out using regionally or locally adapted so-called “isolated or individual solutions” or by means of an individual, time-consuming and usually manual and direct personal data query. Can existing medical health data from healthcare assessment tools provide valid information for the planning of care and care needs in disaster and civil protection?

**Methods:**

Research and analysis of suitable and regularly used care assessment tools in the care sector in Germany.

**Results:**

The healthcare assessment tools cannot be simply combined. Without adaptation they cannot be used at all or only after concerted efforts to interpret the needs for the care of vulnerable groups in a non-clinical context.

**Discussion:**

An improved use of individual medical data in disaster and civil protection offers many advantages in prevention, diagnostics, therapy and care in the context of disaster medicine, and not only from an ethical point of view. Due to the different tactical procedures and care strategies in disaster medicine, the field of acute care must be clearly separated from the area of evacuation and care of vulnerable groups. Currently, only the German Armed Forces (Bundeswehr) offer an internally secured infrastructure that allows all those involved in the care of soldiers to access all important medical data quickly and easily from any location.

## Introduction

Both evacuation planning and post-event medical care depend on valid, up-to-date information. Most locally used or regionally available standalone or isolated solutions are not easily transferable to other regions. They are often based on existing local resources and care structures and do not use standardized assessment algorithms. In addition, the assessment of care needs is not based on existing medical health data, but depends on the amount of information obtained from the patient survey. A standardized data source with up-to-date individual medical data that is available to all parties involved at any time, nationally or even internationally, would be advantageous. One possible data source is the healthcare assessment tools. The following question should be clarified: “Can existing medical data from healthcare assessment tools provide valid information for planning care needs in the event of a disaster?”

### Identification of the demand gap

In the event of a disaster with necessary evacuations, the care of residents with restricted mobility as well as of geriatric residents is in the interests of those affected, of local authorities, health insurance companies, insurance companies and of the responsible authorities and organizations with security tasks (BOS). Although regional and supra-regional evacuation plans have already been drawn up by the relevant authorities (1, 2) stressing to pay attention to people in need of care and possibly limited independence within the affected population groups, there is no draft solution or applicable algorithm (3). Based on the decision of the Federal Constitutional Court of December 16, 2021, which ruled that discrimination on the grounds of disability is inadmissible (1 BvR 1541/20), the legislator is required to take measures to effectively protect these people from any disadvantage in this context as well. In certain constellations, this also obliges the legislator to take concrete action, e.g., in Art. 3 par. 3 sentence 2 of the German Basic Law called Grundgesetz (GG), as well as the involved parties, e.g., in care concepts (4). The World Health Organization (WHO) calls for a standardized and internationally comparable data structure that routinely takes into account data based on the International Classification of Functioning, Disability and Health (ICF) in its draft action paper “Better Health for Persons with Disabilities” in Objective 3 No. 36 and in the measures under 3.2 (5).

For the necessary data collection, it would be ideal to be able to access only existing health data that are released and regulated by the Patient Data Protection Act (PDSG) and the Digital Care and Nursing Modernization Act (DVPMG). Evacuation and care planning is currently carried out by the involved parties through regionally or locally adapted isolated or individual solutions or through an individual, manual and therefore time-consuming and error-prone direct personal data query of the population to be evacuated, including possible contact persons in homes and hospitals. During an operation, this regularly leads to considerable delays in transportation out of the danger zone, which also means that the resources are tied up for too long a time. Furthermore, with the solutions currently available, it is not possible to properly plan the personnel, vehicle and equipment pool required for the evacuation or clearance and the medical care to be provided in the context of vulnerable groups. As a result, patients are sometimes transported in a higher-grade ambulance than actually necessary, which means that it may not be available for more urgent emergency care. This is unacceptable for the waiting patients who depend on this means of rescue and absolutely suboptimal from a (disaster) medical and care perspective, but currently unavoidable without a corresponding database, suitable algorithms and links to data sources in the planning and allocation phase.

In addition, in the event of a disaster or civil protection incident outside of the regular rescue service, there is an initial disproportion between the available rescue teams, rescue resources and the number of people requiring care. This must be compensated for through the targeted use of all resources in order to return as quickly as possible from disaster medicine, including the care of vulnerable groups with increased care and support needs, to regular and individual medical care (6).

## Data research assessment tools

In geriatric medicine, assessment is defined as “a method of evaluating geriatric patients according to functional and social aspects. Restrictions in psychosocial, cognitive, emotional, general physical performance and functional capabilities are examined” (7). For this purpose, before selecting an assessment tool, it is necessary to define the objective (care assessment, risk assessment, etc.), the care area to be applied (hospital, nursing home, home care, etc.), the patient group (geriatrics, pediatrics, specific diseases or care diagnoses) and the user group (nurse, doctor, assistant, etc.) (8). Furthermore, the assessment tools should be highly reliable and valid. The areas of application of assessment tools range from determining care classification, by using diagnostic tests and measurement procedures based on selection criteria up to clinical judgment and clinical reasoning as basic processes for care actions and as screening for an assessment or determination of a need for action (9). The validity of the results about the person in need of care depends on both the quality of the tool and the knowledge and skills of the user (8).

The “special concerns” defined in § 2a of Volume V of the Social Code (SGB V), which must be taken into account, have been specified in a new assessment structure defined by the legislative body in § 14 and § 15 of Volume XI of the Social Code (SGB XI) so that these are used by the National Association of Statutory Health Insurance Funds (GKV-SV), the Federal Medical Service (MD-Bund) and the Medical Service (MD) as an assessment tool for the long-term care requirement. This assessment tool was officially introduced in 2017 under the designation “New Evaluation Assessment (NBA)” (10–13).

## Health data of vulnerable groups

One way of accessing health and care data on vulnerable groups is the existing individual-related care documentation. These documents, which are usually handwritten and not freely accessible, provide an assessment of care needs. To support diagnosis, nationally and internationally developed evaluation tools (risk evaluation) and assessment tools (care needs planning) are used to record care-relevant problems in a structured manner and to transfer them into associated measures or decisions. The extent to which care documentation and the results of the various care assessment tools can be used as a valid source of information for the assessment of the need for care and the necessary medical care requirements in disaster control and corresponding incidents depends on the respective data requirements for a more structured handling of operational planning and non-clinical medical care. As in the care field, information from all six areas of life is required for necessary medical decisions in disaster control (13).

1. Mobility
2. Self-sufficiency
3. Cognitive and communicative abilities
4. Behavioral and psychological problems
5. Dealing with disease-specific/therapy-related requirements
6. Organization of everyday life and social contacts

In order to be able to make a statement regarding the assessment content and results, a literature and web search was carried out prior to the elaboration. Then the following care needs planning tools (assessment tools) and disease severity classifications relevant to preclinical care were selected for closer examination (Table 1). A decisive criterion for the selection of the instruments was the need for a high degree of reliability, standardization, usability and validity and that measures and scores collected with instrument as well as the assessment results should ideally cover all six these domains of life. Pure risk assessment (falls, pressure sores, etc.) or screening instruments (dementia, COPD, pain, etc.) were not included in the detailed analysis. In addition, it had to be checked if planning tools for disaster control and/or civil protection were already in existence or being developed.

**Table 1:** Overview of the included care assessment tools and their information content for the six areas of life.

	Mobility	Self-sufficiency	Cognitive and communicative abilities	Behavioral and psychological problems	Dealing with disease-specific/therapy-related requirements	Organization of everyday life and social contacts	Miscellaneous parameter
ICD/ICF	++	++	++	+	++	+	Body functions Body structure Activities and participation Environmental factors Personal factors
RAI	++	++	++	++	+	++	Identification Information Hearing, Speech, and Vision Cognitive Patterns Mood Behavior Preferences for Customary Routine and Activities Functional Abilities and Goals Bladder and Bowel Active Diagnoses Health Conditions Swallowing/Nutritional Dental Status Skin Medications Special Treatments, Procedures, and Programs Restraints and Alarms Participation in Assessment and Goal Setting
FACE	+	+	+	+	+	++	Health status (general state of health, multisystemic problems) Risk management (e.g., risk of falling) Quality of life (subjective assessment) Sensory abilities Medication
PAS	++	++	+	+	+	+	Resources and strengths of the patient Care needs Continence Pain Nutrition
NBA	++	++	+	++	+	++	Eating and drinking Continence Posture Mobility Day and night rhythm Dressing and undressing Body temperature Personal hygiene Avoiding danger Communication Contact with others Sense of norms and values Everyday activities Leisure activities Learning ability
ePA/epa	+	+	+	+	+	+	Movement Communication/Interaction Personal hygiene and dressing Sleeping Nutrition Breathing Excretion Pain Cognition/awareness Decubitus/wound

ICD, International Statistical Classification of Diseases; ICF, International Classification of Functioning, Disability and Health; RAI, Resident Assessment Instrument; FACE, Functional Assessment of the Care Environment for Older People; PAS, Care Dependency Scale; NBA, New Evaluation Assessment; ePA, Result-oriented nursing assessment; epa, efficient nursing analysis; I.D.A., Information-Documentation-Assessment; 0, Information is not assigned; +, Information is partially assigned; ++, Information is completely assigned.

Due to a lack of data, it is not possible to make a reliable statement about how often individual healthcare assessment tools are used or applied in care facilities in Germany.

No information with empirical values could be found on the use of care assessment tools in civil protection.

## WHO classifications ICD and ICF

The International Statistical Classification of Diseases (ICD) is published by the WHO and provides the basis in Germany for billing in hospitals and medical practices, for the general documentation of diseases and health problems and for electronic data exchange within the medical sector (14). In Germany, the Federal Institute for Drugs and Medical Devices (BfArM) has been commissioned by the Federal Ministry of Health (BMG) to review these classifications, adapt them to German conditions and develop them further in international working groups. The unmodified English version of the ICD-10-WHO is mainly used in Germany for mortality coding. The ICD-10-GM (German Modification) version 2024 has been available since January 1, 2024 and is to be used for morbidity coding. ICD-11 came into effect in Germany on January 1, 2022. Initially it was established for a transition period of 5 years (the so-called quality assurance process); its mandatory use being planned from January 1, 2027. The reasons for revising the ICD arose from the need to be able to classify special circumstances in a more differentiated way than before and to adapt the classifications to the needs of digitized healthcare systems (14).

The ICD-10 and the International Classification of Functioning, Disability and Health (ICF) complement each other and provide a comprehensive picture of the health of an individual or a vulnerable group. The ICF is used across disciplines and countries as a uniform and standardized language to describe a person’s functional state of health, disability, social impairment and relevant environmental factors. The bio-psycho-social model on which the ICF is based on means that there is primarily no deficit-oriented classification (consequences of illness), but rather a resource-oriented classification of the components of health: body functions, body structures, activities and participation (involvement) as well as environmental factors. This rather neutral perspective enables universal applicability to all people and not only to people with disabilities or limitations (15).

## RAI

The Resident Assessment Instrument (RAI) is a tool used to carry out a comprehensive assessment of the current state of health and individual needs of residents in care facilities. It is used by a team of care professionals, including nurses, care assistants and social workers. The RAI supports the mapping of the need for care and the associated problems (16).

The RAI consists of three modules. The Minimum Data Set (MDS) as a structured client assessment form, the Resident Assessment Protocols (RAPS) as so-called clarification aids for a total of 18 geriatric care problem areas and the trigger systems, which alert the caregiver to clinical problem areas (17).

As a rule, the first application (documentation in the individual resident file) of the RAI takes place when a resident is admitted to the care facility in order to establish a starting point for individual care planning. The RAI is then carried out again regularly in accordance with the facility’s guidelines and legal requirements. The frequency of these assessments can be quarterly, unless there are significant changes in a resident’s health status that necessitate an early review and update (18, 19).

## FACE

Functional Assessment of the Care Environment for Older People (FACE) is a tool for care planning in long-term care. FACE was developed back in the 1990s. The system is designed as a modular system and can be used by different professional groups to be put together according to the purpose and intention of the application. The information collected can be used to decide on care objectives and measures and to determine the distribution and assumption of tasks (17). In addition, the individual medical history as well as care and therapy histories can be summarized and visualized.

The assessment with FACE covers six different areas:

- Psychological area (aspects of psychological well-being, signs and symptoms of psychological problems, cognitive functions, behaviors, personality, beliefs, values and attitudes).
- Physical area (general presentation of the health situation, aspects of physical conditions, illnesses, impairments and disabilities).
- Area of activities of daily living (self-care, household management, mobility inside and outside the home).
- Area of human relationships (interpersonal relationships, social network and family).
- Area of social living conditions (housing and living conditions, individual employment, financial circumstances).
- Area of individual risks (20).

## PAS

The Care Dependency Scale (PAS) is a standardized instrument for assessing the need for care, which is used both in hospitals and in a slightly adapted form in geriatric care. In 15 characteristics (items), each with five gradations, from completely dependent to completely independent, patients are systematically assessed with regard to basic needs and their level of care (in)dependency is evaluated.

Patients are assessed by healthcare professionals or caregivers based on their interactions and activities with the patient (17).

## NBA

The so-called New Evaluation Assessment (NBA) was created in 2008 as a prototype of an innovative assessment procedure as part of a pilot project that was jointly developed and presented by the Institute for Nursing Science at Bielefeld University (IPW) and the Medical Service of the Westphalia-Lippe Health Insurance Fund (MDK WL). As part of the care reform and the second Care Strengthening Act (PSG II), it replaced the old guidelines for assessing the need for care in 2017 and has since been used as the basis for care degree classification. The main purpose of this instrument is to assess the need for care in a standardized way in order to determine benefit entitlements. However, the results of the assessment can also be used for individual care planning. It not only covers physical limitations, but also cognitive or psychological impairments and behavioral problems that require special support. The benchmark of the NBA evaluates the degree of independence in carrying out activities and organizing the six areas of life (13).

## ePA/epa

Result-oriented nursing assessment (ePA) as well as efficient nursing analysis (epa) are key figure-based assessment instruments based on nursing science for the evaluation and planning of nursing services in various care areas. The various versions of the ePA include acute care (AC), long-term care (LTC), mental health (PSYC), pediatrics (KIDS) and home care (HC). The fully standardized screening instrument AcuteCare (AC) was specifically developed to measure and assess changes in patient conditions and abilities. The 50 items, which are divided into 10 categories, record the necessary data according to the WHO ICF system. The patient’s abilities according to the ICF are recorded in the areas of activity and participation, while the patient’s condition according to the ICF is recorded in the area of physical functions (21, 22).

In epaSCORING, 10 ePA items are assessed. Depending on the instrument, the ePA scoring system is used to create the Self-Nursing Index (SPI), the Independence Index (SSI) or the Self-Care Index (SFI). This allows the user to visualize the patient’s need for care and suggests decision-making aids for care (21).

These tools and classifications play an important role in healthcare and long-term care to understand health conditions, assess care needs and improve the quality of care. A list of the individual contents of the current version of epaAC was only available to a very limited extent during the research and was not publicly accessible.

## I.D.A

I.D.A—“Information-Documentation-Assessment” is a web-based application in which important information for the care process can be combined. This tool, which was published in 2021, can be used to identify in time changes due to illness and age related changes, as well as to plan tailored support measures and ensure appropriate care in all areas of life.

The corresponding web app was developed as part of the research project “Assessment of healthcare and nursing needs of people with intellectual and/or multiple impairments” (EIBeMeB) at the Ostfalia University of Applied Sciences, Faculty of Healthcare in Wolfsburg. The project was funded by the European Regional Development Fund (ERDF) and with funds from the state of Lower Saxony—Stronger Developed Region (SER) category.

Care facilities that already use I.D.A report an improved and more detailed collection of information regarding the care and health needs of the residents entrusted to them (23, 24). The I.D.A. is the only assessment tool that provides a very comprehensive density of data in all six areas of life and beyond.

## Discussion

From both an ethical and tactical perspective, better use of data offers many advantages in terms of prevention, diagnostics, treatment and care in disaster medicine. By using data, the care, support and necessary treatment of those affected can be ensured as far as possible, even away from their familiar surroundings, without information gaps. An unbalanced relationship between opportunities and risks or strict avoidance of the risks of data use (e.g., through excessive data protection) is unethical in the long run (25). As a result of their training, helpers are mainly trained in acute care, which only includes very superficial nursing and care topics. For the care of vulnerable groups, more in-depth training and preparation is required. This poses a major challenge for the medical task force to consider the needs of both sides from an ethical (no discrimination) and general medical point of view (ensuring the supply of medication and, if necessary, care needs).

If an assessment tool is used, the quality and usability of the data depend on the assessment tool used, the experience of the specialist carrying out the assessment and the aim of the assessment. The numerous, sometimes insufficiently valid assessment tools (not widely in use in care settings), standardized in a very limited way or written in a foreign language are too rarely used again outside the usual reassessment times. Like this, certain data can be obsolete and do not represent the current state of health of those affected, which is necessary for disaster control. Furthermore, several instruments cannot be easily combined, as they were each developed either with the aim of designing a care process or even just assessing a specific situation. Consequently, they cannot be used at all or only with a great effort to interpret them for the needs of preclinical or non-clinical contexts of disaster medicine. Furthermore, comprehensive data availability, including the current status of health data before and during a medical mission, cannot currently be guaranteed.

In order to access the assessment information provided by the care assessment tools, it is necessary to digitize the handwritten care documents or to access the electronic care documents of the person concerned. Due to the non-standardized access to health data, it may not be possible to meet the requirements of the PDSG. An alternative data source that still needs to be tested is the electronic health record, which will be mandatory in Germany in a few weeks. All data protection requirements can be met by retrieving data via the specified data interfaces.

The German Armed Forces “Patient Evacuation Coordination Center” (PECC) project shows that digitization can work in healthcare. Here, the step towards digitization has already been taken in internal healthcare. Thanks to the secure infrastructure provided by BWI (the German Armed Forces’ IT service provider), all members of the German Armed Forces involved in the care of troops (doctors, nurses) can access all important medical data within the electronic German Armed Forces’ health record (eGABw) quickly and easily from any location. Furthermore, the PECC project has created an opportunity to manage the transfer of sick or injured soldiers or civilians from countries of deployment to the German Armed Forces hospital best suited to their care. The software also provides a background check of the medical data for missing information or inconsistencies (26).

## Conclusion

- From an ethical point of view, the use of existing data offers many advantages for the care, support and necessary treatment of those affected can be ensured as far as possible, even away from their familiar surroundings, without information gaps. An unbalanced relationship between opportunities and risks or strict avoidance of risks is unethical in the long run.
- A preferred approach would be to access only existing health data that are released and regulated by the Patient Data Protection Act (PDSG) and the Digital Care and Nursing Modernization Act (DVPMG).
- Early risk identification, analysis and assessment, correct resource assessment, provision and allocation lead to increased security and quality of supply, especially for vulnerable population groups in the event of evacuation.
- The assessment tools cannot be easily combined, which limits their use in the non-clinical context of disaster medicine without adaptation.

### Recommendations

As already mentioned above, it would be ideal for the necessary collection of data to be able to access only existing health data that is released and regulated by the PDSG and the DVPMG. The healthcare assessment tools provide a very good basis for data collection for their respective areas. However, none of the assessment tools fully provide the necessary medical and nursing data that would be helpful for external care in the event of a disaster. A standardized platform with access to all necessary data could be the electronic health record, which will be available to all people with statutory health insurance in Germany from January 15, 2025. Here, however, it would be necessary to expand the emergency data set it contains to include the data still required for care and any other needs in a standardized way. Access to the emergency data set is possible for authorized medical personnel at any time via standardized interfaces.

For standardized data retrieval in the electronic health record, it makes sense to create a flexible data retrieval interface that can be integrated into the operational control and support software systems used. Intensive data retrieval tests and scenario-dependent planning simulations based on retrieved health data should be implemented in further research projects.

### Miscellaneous

The present work was carried out in fulfillment of the requirements for the degree of “Dr. rer. medic.” at the University of Witten/Herdecke, Chair of Geriatrics.

## Data Availability

The original contributions presented in the study are included in the article/supplementary material, further inquiries can be directed to the corresponding author.
